# Effects of the level and instability of self-esteem on help-seeking and its orientation

**DOI:** 10.1093/geroni/igaf122.2988

**Published:** 2025-12-31

**Authors:** Jun Nakahara

**Affiliations:** Chukyo University, Toyota, Aichi, Japan

## Abstract

Some older adults do not seek or even refuse help when needed or when help is available. Therefore, it is important to examine the factors of help-seeking behavior and its orientation among older adults, particularly self-esteem. Previous theoretical assumptions show that individuals with high self-esteem and instability would estimate the ego-threat associated with help-seeking as high and suppress help-seeking; conversely, those with low self-esteem and high instability would be motivated to improve their self-evaluation and tend to productively cope with the task, which would promote help-seeking. This study aims to test these hypotheses. Data were obtained from 138 older adults through a one-week diary survey using the experience sampling method. The seven-day intra-individual standard deviation was used for the self-esteem instability index, and the mean was used for the level index. Results of the multiple regression analysis for the number of help-seeking behaviors in seven days revealed that the interaction term for the level and instability of self-esteem was significant. The simple slope test showed that the slope of self-esteem instability was positive and significant when the level of self-esteem was 1-SD below. Conversely, it was negative and not significant when the level of self-esteem was 1-SD above. There was no relationship between self-esteem indices and help-seeking orientations. This result suggests that some older adults were motivated to improve their self-evaluation and tended to productively cope with tasks, which promoted help-seeking. The lack of a relationship between help-seeking orientations and self-esteem suggests that the psychological process regarding help-seeking is unconscious.

